# Assessing genetic diversity in critically endangered *Chieniodendron hainanense* populations within fragmented habitats in Hainan

**DOI:** 10.1038/s41598-024-56630-0

**Published:** 2024-03-24

**Authors:** Li Zhang, Hai-Li Zhang, Yukai Chen, Mir Muhammad Nizamani, Tingtian Wu, Tingting Liu, Qin Zhou

**Affiliations:** 1 Guizhou Normal University Museum, Guizhou Normal University, Guiyang, 550001 China; 2 Sanya Nanfan Research Institute, Hainan Yazhou Bay Seed Laboratory, Sanya, 572025 China; 3 Ministry of Education Key Laboratory for Ecology of Tropical Islands, College of Life Sciences, Hainan Normal University, Haikou, 571158 China; 4 Department of Plant Pathology, Agricultural College, Guizhou University, Guiyang, 550001 China; 5 Hainan Academy of Forestry, Hainan Academy of Mangrove, Haikou, 570228 China

**Keywords:** *Chieniodendron hainanense*, Endangered plants, GBS, SNP, WPESP, Ecology, Ecological genetics

## Abstract

Habitat fragmentation has led to a reduction in the geographic distribution of species, making small populations vulnerable to extinction due to environmental, demographic, and genetic factors. The wild plant *Chieniodendron hainanense*, a species with extremely small populations, is currently facing endangerment and thus requires urgent conservation efforts. Understanding its genetic diversity is essential for uncovering the underlying mechanisms of its vulnerability and for developing effective conservation strategies. In our study, we analyzed 35 specimens from six different populations of *C. hainanense* using genotyping-by-sequencing (GBS) and single nucleotide polymorphism (SNP) methodologies. Our findings indicate that *C. hainanense* has limited genetic diversity. The observed heterozygosity across the populations ranged from 10.79 to 14.55%, with an average of 13.15%. We categorized the six populations of *C. hainanense* into two distinct groups: (1) Diaoluoshan and Baishaling, and (2) Wuzhishan, Huishan, Bawangling, and Jianfengling. The genetic differentiation among these populations was found to be relatively weak. The observed loss of diversity is likely a result of the effects of natural selection.

## Introduction

The conservation of biodiversity is crucial for sustaining the stability and resilience of ecosystems. Genetic diversity constitutes a pivotal element in the long-term persistence of species, particularly for those facing extinction risks. *Chieniodendron hainanense* represents a rare and imperiled plant species characterized by extremely small populations (WPESP) and is endemic to the fragmented habitats of Hainan, China^1^. Owing to the adverse consequences of habitat fragmentation, the genetic diversity of *C. hainanense* populations warrants significant attention in conservation initiatives. Comprehending the genetic architecture and diversity of these populations enables the development of suitable management approaches and facilitates species restoration efforts^2^.

The genetic structure and diversity of a species play critical roles in their ability to withstand adverse environments and evolve over time. Studying the genetic makeup of wild plants with extremely small populations can provide insights into their evolutionary history, population dynamics, and response to environmental changes, enabling the development of effective conservation strategies^3^. Such research is also essential for advancing DNA sequencing technology and conservation biology^4^.

Habitat fragmentation and overuse of resources have greatly impacted species’ genetic diversity, survival, adaptability, and biodiversity^5^. Habitat fragmentation reduces genetic diversity by isolating populations and restricting gene exchange, leading to an increase in genetic drift and inbreeding depression^6,7^. The reduced genetic diversity weakens the ability of species to adapt to environmental changes and increases the risk of species extinction^8^. Habitat fragmentation also reduces the number of plant populations, increases spatial isolation, and hinders the maintenance of population genetic diversity by disrupting dispersal, gene exchange, and inter-species interactions^9^.

Wild plants with extremely small populations (WPESP) are endangered species under national key protection and require urgent rescue efforts.Their population sizes are smaller than the minimum viable and are usually distributed in a narrow area, making them highly vulnerable to extinction^10,11^. The Chinese government has launched the National Project Plan for the Rescue and Protection of Wild Plants in Extremely Small Populations (2011–2015) to protect 120 WPESP species, most of which are endemic to China and have significant ecological, scientific, cultural, and economic value^12^. The loss of biological and genetic values of WPESP due to their extinction can have significant adverse effects on human society and the ecosystem^13^. Therefore, it is crucial to prioritize research on WPESP conservation in current biodiversity conservation studies in China.

*Chieniodendron hainanense* is a second-class national key protected wild plant and a unique member of the Annonaceae family Chieniodendron genus in China. The species is an evergreen tree that grows up to 16 m tall with a DBH of about 50 cm, and its distribution is limited to the Guangxi Zhuang Autonomous Region and some areas of Hainan Province. Habitat fragmentation due to human activities such as logging has drastically reduced the distribution area of *C. hainanense*, and the wild resources of its population are scarce. Field surveys have shown that the existing populations of *C. hainanense* are mainly distributed in nine primary forest areas dominated by fragmented secondary rainforests in Hainan, with many original populations already disappeared^14^. The species has poor self-renewal ability and is highly sensitive to external disturbances, making its wild resources already endangered. Although some research reports have focused on the population structure, dynamic characteristics, leaf morphology, petal nodules development, and functional biochemical activities of *C. hainanense*, very few studies have investigated its endangerment mechanisms at the molecular level. To develop targeted and comprehensive conservation strategies, it is necessary to understand the causes of its endangerment, including its genetic diversity, and related components^15^.

In this investigation, our objective is to evaluate the genetic diversity and population structure of *C. hainanense* across its fragmented habitats in Hainan, utilizing genotyping-by-sequencing (GBS) and single nucleotide polymorphism (SNP) techniques. Through the analysis of 35 specimens from six discrete cohort groups, we will scrutinize patterns of genetic variation, differentiation, and inbreeding both within and among the populations. The outcomes of this research will yield valuable insights into the conservation status and management of *C. hainanense*, promoting the formulation of efficacious strategies for the preservation and restoration of this critically endangered species. Additionally, our findings will augment the understanding of the wider implications of habitat fragmentation on genetic diversity in plant populations and inform conservation endeavors for other threatened plant species inhabiting fragmented ecosystems.

## Materials and methods

### Study area

**Figure 1:**
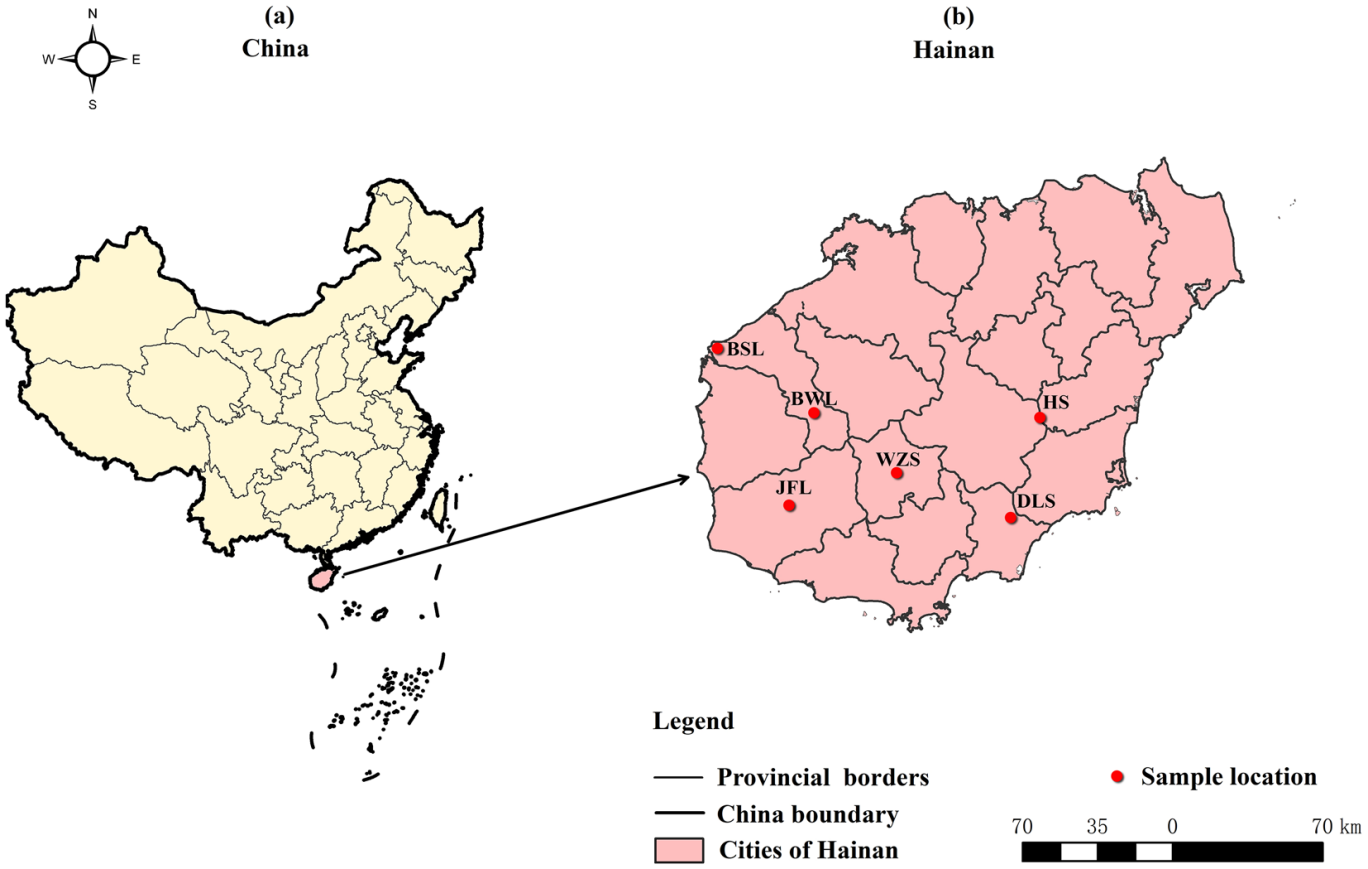
Distribution of *C. hainanense* on Hainan Island and sample collection information map. (**a**) Location of Hainan Island in China, (**b**) Distribution of *C. hainanense* on Hainan Island.

Hainan Island (E108°37′–111°03′, N18°10′–20°10) is situated in the southern part of China, on the northern edge of the tropics (Fig. 1). It has a tropical monsoon climate and a mild climate with long summers and short winters^16^. The annual average temperature ranges from 22 to 27 °C, and the island has abundant water. Hainan Island is an important distribution area for China’s monsoon forests and tropical rainforests^17^. The main forest vegetation types in Hainan Island are tropical rainforest, evergreen broad-leaved forest, coniferous forest, and plantation forest. Hainan Province has the largest tropical rainforest in China, with rich flora and fauna resources, covering 42.5% of the total tropical area of the country^18^. The critical areas for biodiversity conservation in my country, “National Of the 120 species of wild plants in extremely small populations identified in the Plan for the Rescue and Protection of WPESP (2011–2015). Hainan has 24 key protection targets. Yunnan and Hainan are critical areas for the protection of WPESP ^19^.

### Materials

During 2019–2021, 35 *C.hainanen* samples were continuously collected from Hainan Island. They were divided into six regions according to the geographical origin of the samples, namely, the Bawangling population (samples BWL_1-BWL_8), the Jianfengling population (JFL_1-JFL_5), the Luoshan population (DLS_1-DLS_10), Huishan population (HS_1-HS_6), Wuzhishan population (WZS_1-WZS_3), Baishaling population (BSL_1-BSL_3). The division of regional populations, latitude, longitude, and the number of samples are shown in Table 1.

**Table 1:** The list of information for *C. hainanense* sample collection on Hainan Island.

Area code	Location	Longitude	Latitude	Sample	Number of samples
BWL	Bawanglin	109°07′–109°12′E	19°04′–19°07′N	BWL_1-BWL_8	8
JFL	Jianfengling	109°39′–109°48′E	18°41′–18°46′N	JFL_1-JFL_5	5
DLS	Diaoluoshan	109°19′–109°56′E	19°03′–19°04′N	DLS_1-DLS_10	10
HS	Huihan	110°08′–110°09′E	19°03′–19°04′N	HS_1-HS_6	6
WZS	Wuzhishan	108°50′	18°46′N	WZS_1-WZS_3	3
BSL	Baishalin	109°14′E	19°11′N	BSL_1-BSL_3	3

### Methods

Firstly, DNA was extracted from 35 *C. hainanense* leaf samples, and the quality and concentration of the extracted DNA were tested before being sent to Hangzhou Lianchuan Biotechnology Co. After sequencing was completed, the SNPs of the *C. hainanense* genome were mined. Based on these SNPs, the phylogenetic tree analysis of *C. hainanense* was obtained using the neighbor-joining algorithm of MEGA software. Principal component analysis (PCA) was then performed on *C. hainanense* populations based on the SNPs. Additionally, the population structure of all samples was analyzed using admixture software to obtain the distribution of genetic material in different populations of *C. hainanense*. Finally, genetic distances among all samples were calculated based on the SNPs. The detailed method is described in the paper by Chen et al.^20^.

#### Enzyme digestion protocol design

Our simplified genome digestion scheme selected according to other research methods is as follows, the restriction enzyme combination of HaeIII + Hpy166II was selected. The ‘Insert Size’ was selected as ‘550–600 bp’^21^.

#### Sequencing quality control

The Raw data (the number of reads in the original downstream data) generated by sequencing is pre-processed by quality filtering to obtain CleanData. The specific processing steps are as follows: (1) remove the adapter, (2) remove the reads containing N (N means the information of bases cannot be determined) with a proportion of more than 5%, (3) remove the low-quality reads (the number of bases with quality value Q <  = 10 accounts for more than 20% of the whole reads), (4) count the raw sequencing volume, effective sequencing volume, Q20 (the proportion of bases with quality values greater than or equal to 20, sequencing error rate less than 0.01), Q30 (the proportion of bases with quality values greater than or equal to 30, sequencing error rate less than 0.001), GC means guanine (G) and cytosine (C)content, and perform a comprehensive evaluation.

#### Comparison of consistency sequences

We used Burrows-Wheeler aligner (BWA) software to match the sequencing data to the consistent sequences obtained from reads clustering. Since the reference used is the consistency sequence obtained from reads clustering, the matching rate will vary somewhat between samples.

#### Variation detection and SNP statistics

After comparing the data with the concordant sequences, we used Genome Analysis Toolkit (GATK) and SAMtools software for variant detection, retaining the SNPs that were consistently output by both software as reliable loci. We further processed the SNP data by filtering them based on MAF > 0.05 and data integrity > 0.8 and retained the SNPs with polymorphisms among them. The final filtered SNPs were input to the subsequent evolutionary analysis.Based on the obtained SNP data, we analyzed the genetic evolution and structure of the population using the differences in genetic information among the samples of *C. hainanense*, including the phylogenetic relationships among the samples, population structure, principal component analysis (PCA), and relatedness among the samples. The following part of the analysis involves grouping samples, and the 35 samples were divided into six groups according to species for analysis.

#### Data statement

This study strictly adheres to the guidelines and legal regulations of the National Forestry and Grassland Administration, including experimental research and field studies on plants, as well as the collection of plant materials, among others. Voucher specimens of wild plants have been stored at Hainan Normal University, located at 99 Longkunnan Road, Qiongshan District, Haikou City. Voucher number is *C. hainanense* 001–030.The collection of plant specimens for this study has been authorized by the reserve’s forestry bureau.

## Results

### Simplified genome quality inspection of *C. hainanense*

A total of 49.16 GB of raw data were obtained through genotyping-by-sequencing (GBS) on 35 *C. hainanense* samples (Supplymentary information). After removing the base information that could not be determined in the adapter sequence, the remaining 48.52 GB of high-quality sequencing data (Clean data), the average of each sample was 1.39 GB. In the simplified genome of *C. hainanense,* the average Q20 was 96.71%; the average Q30 was 90.97%. The average ratio (GC content) of guanine (G) and cytosine (C) is 40.60%. The results indicated that the sequencing quality of Q20 and Q30 was high, the GC distribution was reasonable, and the sequencing information was reliable.

### *C. hainanense* SNP site mining

In this study, the heterozygosity of the six populations of *C. hainanense* ranged from 10.79% to 14.55%, and the average heterozygosity is 13.15%, which shows the genetic diversity of *C. hainanense* trees is low (Table 2).

**Table 2:** SNP Statistical results.

Population code	SNP number	Heter LociNum	Homo LociNum	Hetloci-ratio (%)
BWL	181002	25265	155737	13.93
JFL	171854	23417	148437	13.45
DLS	183868	25185	158683	13.59
HS	199196	25077	174119	12.56
WZS	190463	27590	162873	14.55
BSL	172311	18612	153699	10.79

### Genetic evolution and population analysis

#### Phylogenetic evolutionary tree

**Figure 2:**
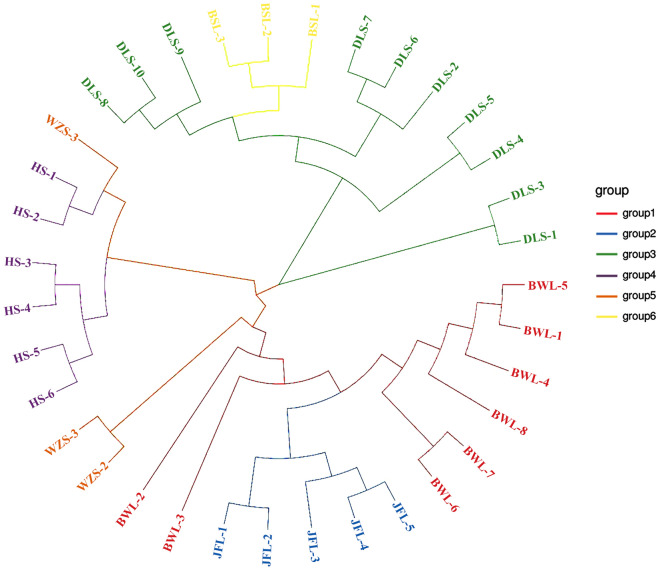
The neighbor-joining cluster of *C. hainanense* in different population.

We divided the 35 *C. hainanense* samples roughly into two general taxa, which can be further subdivided into several subgroups. Group I mainly comprise resources from Diaoluoshan (DLS) and Baishaling (BSL), while Wuzhishan (WZS), Huishan (HS), Bawangling (BWL), and Jianfengling (JFL) are clustered into group II (see Fig. 2). The small subgroups clustered in group I were further divided into two smaller subgroups, indicating a relatively significant genetic relationship between each other in this larger group. The aggregation of samples in the second group is relatively chaotic, suggesting that there is less genetic relationship among the individuals in this population. From this perspective, although there is some geographical isolation between *C. hainanense* resources from different areas, there is a direct and necessary relationship between clustering based on genetic distance and their geographical origin.

### Analysis of population genetic structure

**Figure 3:**
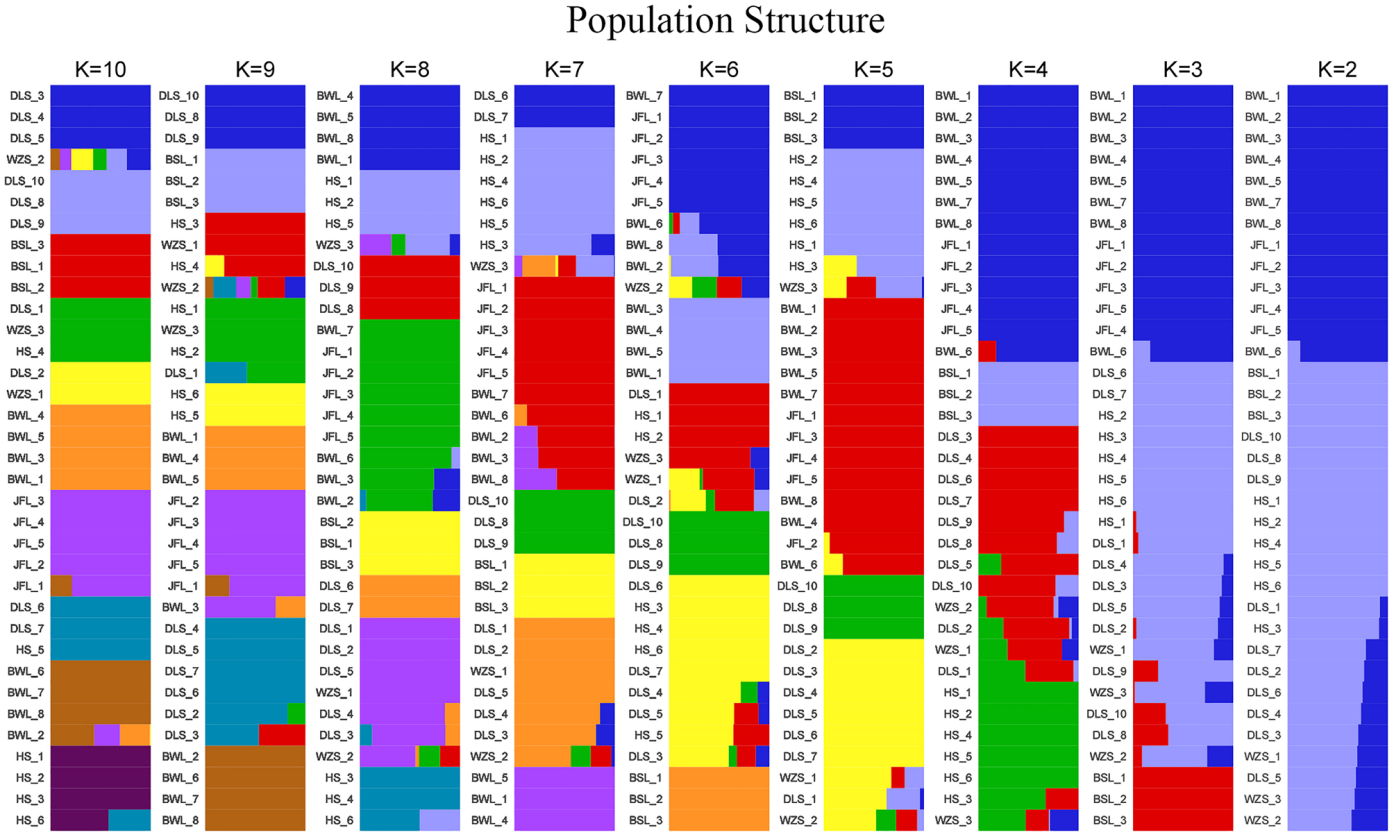
The population structure analysis on* C. hainanense.*

**Figure 4:**
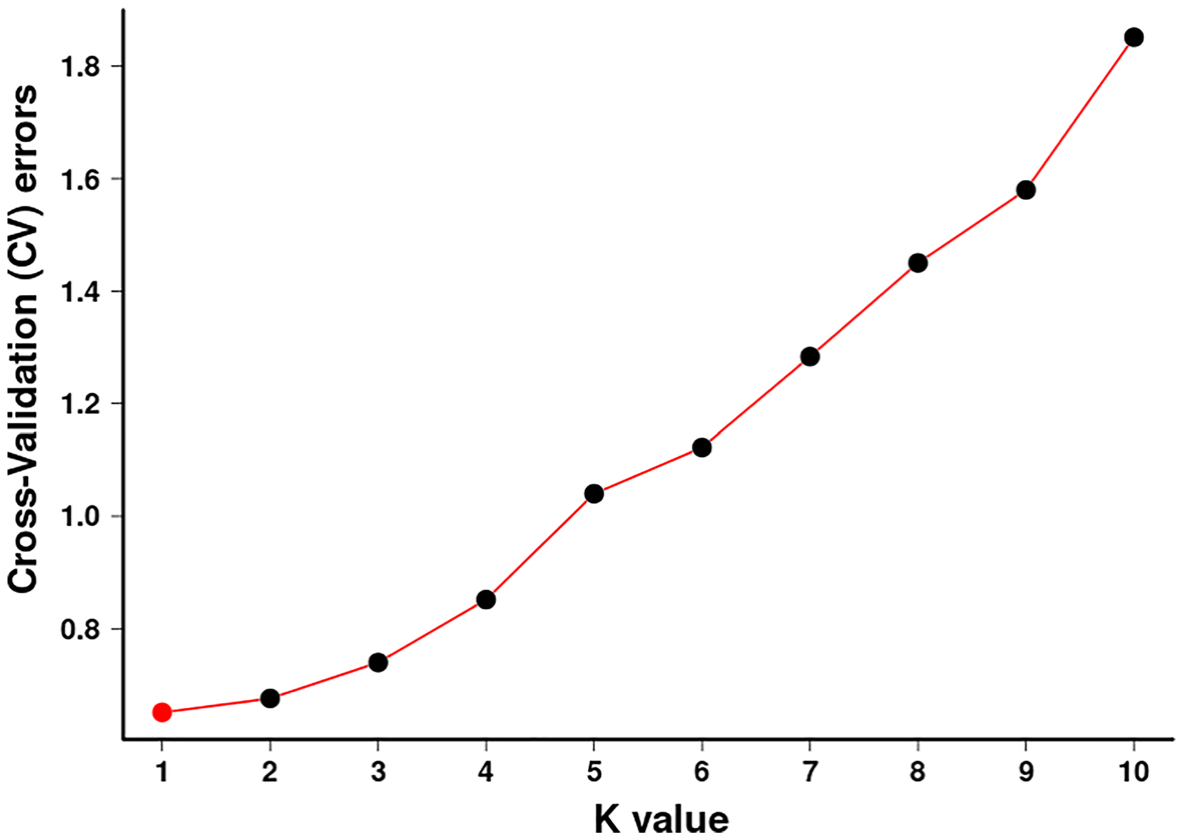
K selection of population structure.

When K = 2, the samples from group 1 appear almost dark blue, and the samples from group 2 appear almost light purple. Upon dividing the samples into two subgroups, the samples from JFL and BWL were clustered into group 1, and the remaining samples were clustered into group 2 (Fig. 3). In the cross-validation (CV) error plot (Fig. 4), CV error reached its minimum value when K = 1, indicating that the genetic differences among *C. hainanense* samples were relatively small, and their genetic relationships were close. Thus, it can be preliminarily inferred that the six *C. hainanense* populations in Hainan Island originated from the same ancestor and had undergone more gene exchanges. In the table of genetic differentiation coefficients among populations (Table 3), the F_st_ values among the six *C. hainanense* populations were between -0.09648 to 0.076729. There was a moderate degree of genetic differentiation (0.05 < F_st_ < 0.15) between the two populations of BSL and JFL. Furthermore, the genetic differentiation among other populations was low, indicating that differentiation was not significant (F_st_ < 0.05).

**Table 3:** Genetic differentiation coefficient (F_st_: above diagonal).

	BWL	JFL	DLS	HS	WZS	BSL
BWL		− 0.06459	− 0.01902	− 0.00697	− 0.04823	0.025937
JFL			− 0.03474	0.007746	− 0.02721	0.076729
DLS				− 0.05677	− 0.09648	− 0.05853
HS					− 0.05853	0.013874
WZS						0.042864
BSL						

### Principal component analysis results

**Figure 5:**
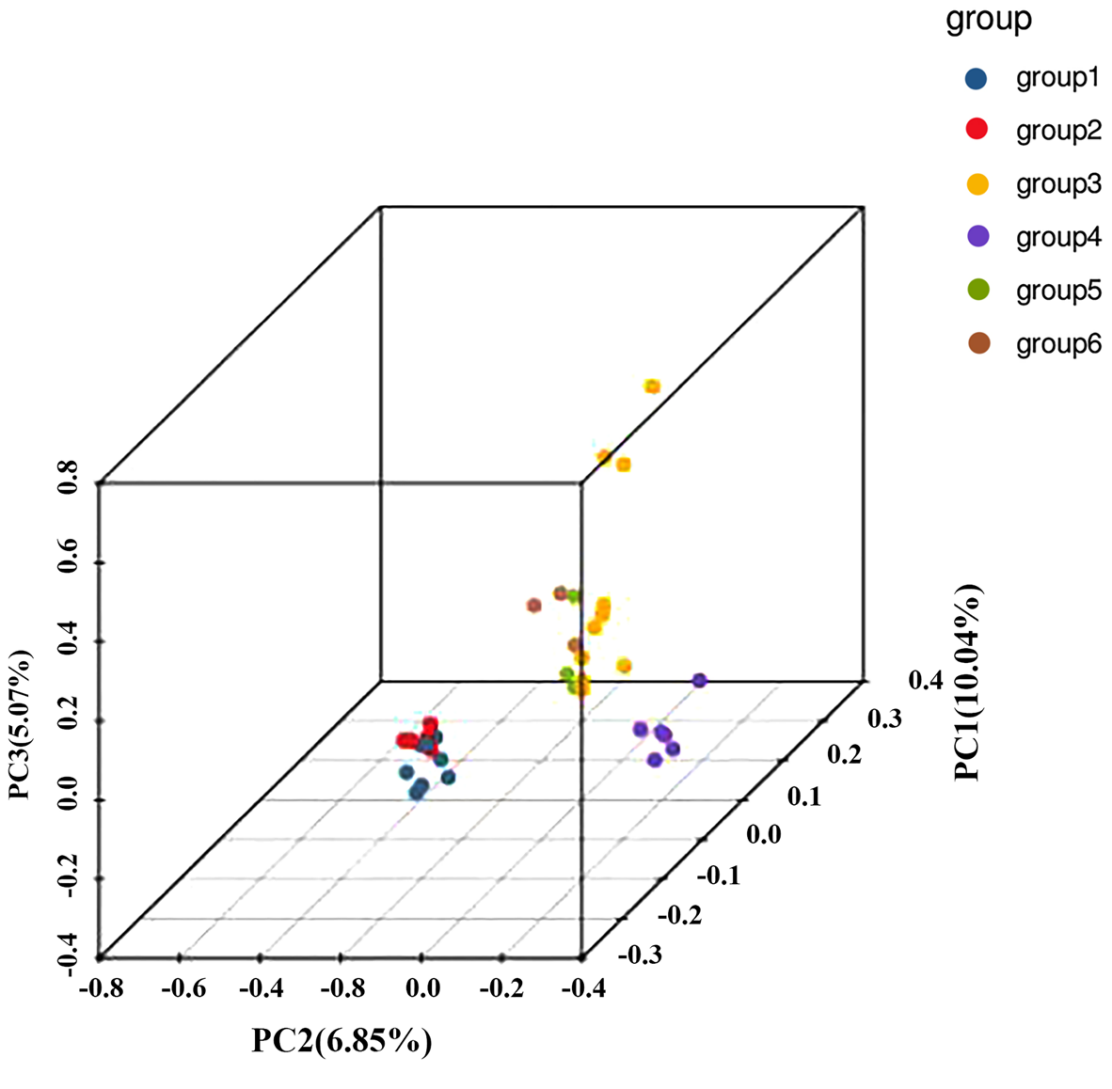
PCA analysis diagram of *C. hainanense.*

Thirty-five clusters of *C. hainanense* were formed, which resulted in four independent clusters. Among these clusters, 13 samples from Jianfengling (JFL_1-JFL_5) and BWL (BWL_1-BWL_8) populations with similar genetic backgrounds, gathered to form cluster 1 (Fig. 5). Six samples of *C. hainanense* from Huishan (HS_1-HS_6) and Wuzhishan (WZS_3) populations, with similar genetic backgrounds, clustered together to form cluster 2. Thirteen samples of *C. hainanense* from Diaoluoshan (DLS_1-DLS_10) and Baishaling (BSL_1-BSL_3) populations, with similar genetic backgrounds, clustered together to form cluster 3. The *C. hainanense* from the Wuzhishan (WZS_1-WZS_2) population was distant from the other three clusters, showing relatively long genetic distances, and so formed a separate cluster 4.

### Analysis of the genetic relationship of *C. hainanense*

**Figure 6:**
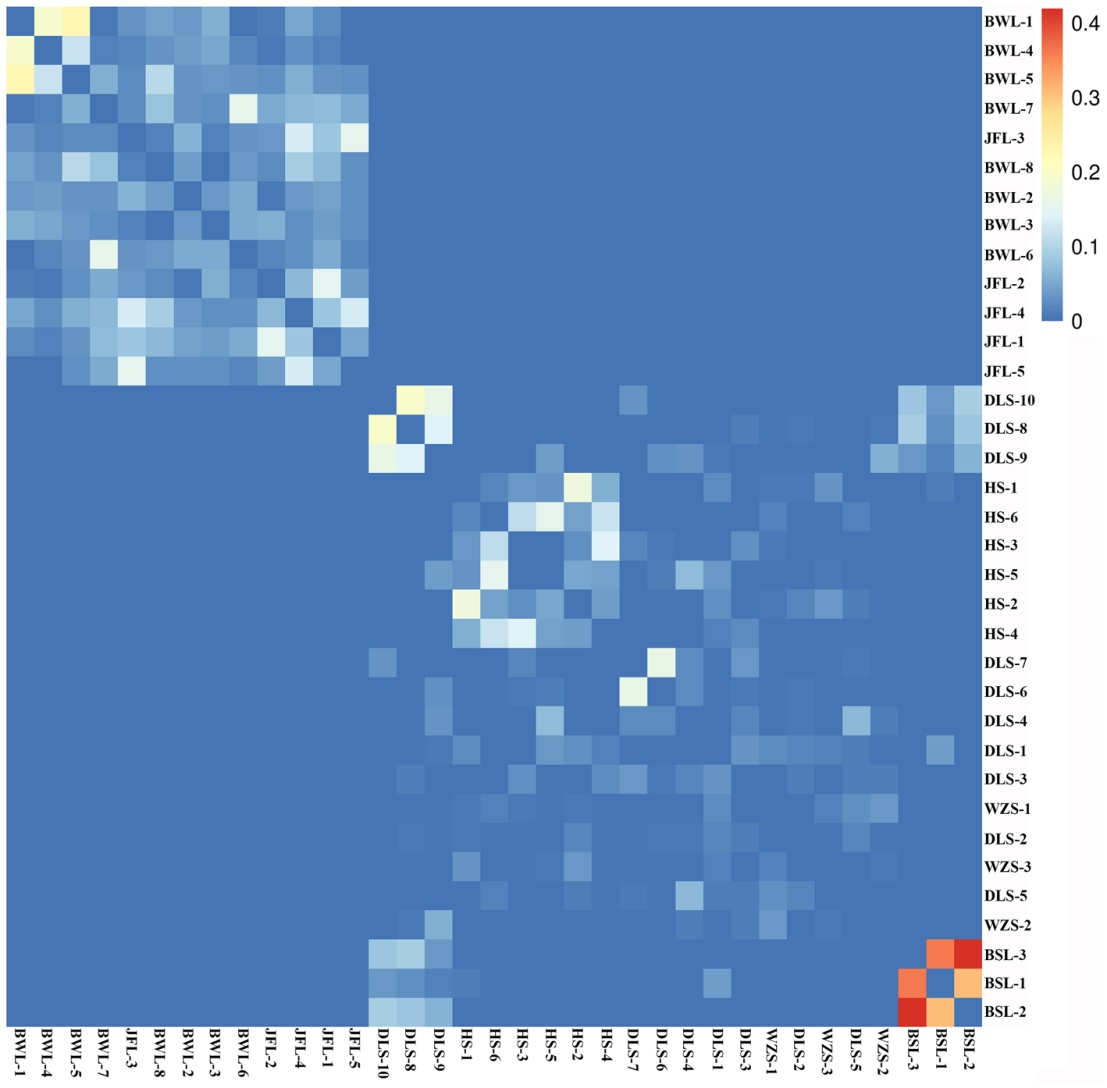
Relatedness of the Bawangling population (samples BWL_1-BWL_8), the Jianfengling population (JFL_1-JFL_5), the Luoshan population (DLS_1-DLS_10), Huishan population (HS_1-HS_6), Wuzhishan population (WZS_1-WZS_3), Baishaling population (BSL_1-BSL_3).

We calculated the relatedness between pairs of all samples based on the SNPs (Fig. 6). Kinship analysis reveals the genetic distance between samples, aiding evolutionary analysis. In the kinship heatmap, redder colors indicate closer kinship between individuals on the horizontal and vertical axes. In contrast, a red heatmap among multiple individuals suggests they may belong to a closely related family group. Conversely, bluer colors indicate more distant kinship between individuals. In the correlation heat map, the correlation coefficients of BSL_1 and BSL_2 were more outstanding than 0.4, indicating that the two samples of Bawangling were very closely related to each other. The correlation coefficients of Bawangling (BWL_1 and BWL_4), Hangluo Mountain (DLS_6 and DLS_9; DLS_7 and DLS_9; DLS_8 and DLS_10), and Huishan (HS_1-HS_6) are just between 0.2 and 0.3, indicating that some genetic exchange still exists between clusters in the case of geographical isolation (Fig. 6).

## Discussion

### Genetic diversity in* C. hainanense*

The genetic diversity of plants is usually influenced by their range, longevity, reproductive systems, seed dispersal mechanisms, and evolutionary history^22,23^. SNP variants are the most significant and extensive type of sequence variation in plant genomes and can be easily identified by sequence alignment. Our study yielded 477,588 high-quality SNPs through screening and filtering. *C. hainanense* has a wide ecological population in the natural wild state, but the seeds require high germination conditions in the natural environment, limiting the population. The natural wild *C. hainanense* is distributed as individuals in fragments in the natural tropical forest. So far, no clusters of communities have been found, so the population density of wild *C. hainanense* is very low, resulting in the population’s weak stress resistance and reproductive ability. Population development is long, and natural recovery is slow.

The low genetic diversity observed in *C. hainanense*, as indicated by heterozygosity values between 10.79 and 14.55%, is a significant concern for the species’ conservation. This finding is crucial given the importance of genetic diversity in a species’ ability to adapt to environmental changes and resist emerging diseases or pests. Studies have consistently shown that higher genetic diversity increases a population’s resilience to environmental shifts^24^. In contrast, low genetic diversity, as seen in *C. hainanense*, can lead to inbreeding depression, a phenomenon where the increased expression of harmful recessive genes in a closely related population results in reduced fitness^25^.

Comparatively, other plant species with higher genetic diversity show greater adaptability. For instance, studies on diverse plant species demonstrate a correlation between genetic diversity and the ability to withstand various environmental stresses^26^. The reduced genetic diversity in *C. hainanense* starkly contrasts with these examples, highlighting its vulnerability to extinction and ecological shifts.

Moreover, the ecological implications of low genetic diversity cannot be understated. A species with limited genetic variation may struggle to fulfill its role in the ecosystem, potentially destabilizing the ecological balance^27^. This is particularly concerning for *C. hainanense*, which, due to its low genetic diversity, might not effectively contribute to the ecosystem’s functionality.

The conservation of *C. hainanense*, a species characterized by low genetic diversity, necessitates a comparative analysis with similar species to understand its unique genetic challenges and inform effective conservation strategies. Studies have shown that genetic diversity is crucial for species’ adaptability and survival. Higher genetic diversity within a population enhances its resilience to environmental changes^26^. When compared to species like *Abies ziyuanensis* or *Taxus contorta*, which are also among the plants with extremely small populations and low genetic diversity as reviewed by Xu (2022), *C. hainanense*’s low genetic diversity puts it at a similar risk of inbreeding depression and reduced adaptability.

Additionally, the impact of habitat fragmentation, as seen in *C. hainanense*, is a common threat to many species and has been extensively studied. How habitat fragmentation not only reduces population sizes but also increases spatial isolation, leading to decreased gene flow and genetic diversity^9^. This is evident in *C. hainanense* and is comparable to the situation in other plant species experiencing similar pressures.

Furthermore, the concept of Wild Plant with Extremely Small Populations (WPESP) Xu (2022), applies to *C. hainanense*. These populations often suffer from genetic drift and bottleneck effects, leading to decreased genetic diversity and fitness. The comparative analysis of *C. hainanense* with other WPESP species such as Glyptostrobus pensili and Sinojackia huangmeiensis, which also exhibit low genetic diversity, can provide insights into common patterns and conservation needs.

In light of these findings, conservation efforts for *C. hainanense* must include habitat protection, genetic reserve establishment, and controlled breeding programs. Understanding the genetic status of *C. hainanense* in comparison with similar species is crucial for devising effective conservation strategies. This comparative approach can reveal shared vulnerabilities and potential solutions, facilitating more targeted and efficient conservation efforts.

### Genetic differentiation and genetic structure in *C. hainanense*

Our PCA analysis indicating that PC1, PC2, and PC3 contributed 10.04, 6.85, and 5.07%, respectively, and the distinct clustering of the Wuzhishan population, suggests unique genetic features in this population. This finding aligns with the general utility of PCA in genetic studies, especially in understanding population structures and genetic diversity.

However, it’s crucial to note some of the limitations and challenges associated with PCA in genetic studies. A recent critical evaluation of PCA-based findings in population genetic studies suggests that results from PCA can sometimes be biased and need careful interpretation. This study used a color-based model analogous to SNPs to assess the accuracy of PCA in representing the true distances between different populations or groups. The study concluded that while PCA could be a useful tool for visualizing data and generating hypotheses, it should be used cautiously, especially when drawing far-reaching conclusions about population history or structure from PCA results alone. The study also highlighted that PCA results could sometimes be contradictory or influenced by the experimenter’s choices, such as sample sizes or selection of markers^28^.

In genetic studies, PCA is often used to investigate population structures and migration events. For instance, it has been instrumental in identifying important migration events and understanding the genetic diversity between and within populations. However, the interpretation of PCA results must consider potential distortions or biases that can arise from the methodology itself or the specific data used^29^.

Considering these insights, while your PCA results on the Wuzhishan population provide valuable information about potential unique genetic features, they should be interpreted in the context of these limitations. It’s essential to corroborate these findings with other methods or analyses to ensure a comprehensive understanding of the genetic diversity and population structure of *C. hainanense*. Additionally, further research exploring the specific genetic differences and potential environmental or ecological factors driving these differences would be valuable in understanding the species’ evolutionary history and informing conservation strategies.

The division of 35 *C. hainanense* samples into two primary clusters, as revealed through PCA, K-value selection, and phylogenetic tree analysis, offers valuable insights into the species’ genetic structure. This consistent clustering pattern suggests significant differentiation likely influenced by factors such as historical gene flow, geographical isolation, or habitat fragmentation. These findings align with the principles outlined by various studies in population genetics. For example, the role of geographical isolation in genetic differentiation is well-established, with isolated populations often developing unique genetic characteristics due to limited gene flow and genetic drift^26^. Additionally, habitat fragmentation, a common consequence of human activities, has been shown to significantly impact gene flow and population structure^9^. The observed genetic divergence in *C. hainanense* could also be attributed to different evolutionary pressures or environmental conditions, as environmental variability can drive adaptive genetic changes in populations^30^. Furthermore, the impact of demographic histories such as population bottlenecks or expansions on genetic diversity is widely recognized^31^. The consistency across various analytical methods adds robustness to these findings, highlighting the importance of using multiple approaches to elucidate the complex dynamics of population genetics^32^.

Differences in population genetic structure are an important expression of genetic diversity. A species’ evolutionary potential and ability to withstand adverse environments depends not only on the level of genetic variation within the species but also on the genetic structure of the species^27–29^. Our results indicate that the six populations on Hainan Island are divided into two broad taxa, consistent with the principal component analysis results. A population’s genetic differentiation index (Fst) is an important parameter to measure the degree of genetic differentiation among populations. It can explain the factors that affect the genetic differentiation of populations^30^. The genetic differentiation coefficient among the populations showed that the Fst values among the six *C. hainanense* populations were between − 0.09648 and 0.076729. There is a moderate degree of genetic differentiation (0.05 < Fst < 0.15) between the two populations of Baishaling (BSL) and Jianfengling (JFL). Furthermore, the genetic differentiation among other populations is low, and the differentiation is not noticeable (Fst < 0.05). Therefore, the genetic differentiation among *C. hainanense* populations is weak. The existing gene flow may originate from the genetic exchange between their common ancestor populations and be brought into other populations by other factors such as human factors, animal carrying, or geological factors. In addition, the topography may also affect gene flow in this species. The terrain of Hainan Island is high in the middle and low in the surrounding areas. It rises and descends to the periphery step by step. The cascade structure is prominent, and the terraces, hills, plains, and mountains form a ring-shaped layered landform^31^.

The study of *C. hainanense*’s genetic diversity across various populations, specifically from Wuzhishan, Diaoluoshan, Jianfengling, and Baishaling, reveals significant insights into its population genetics. The notably small genetic distance (Fst = 0.076729) between Jianfengling and Baishaling populations, despite their geographical distance, highlights a potential historical gene flow. This aspect of genetic connectivity across distant populations aligns with findings in the field of plant population genetics^9,33^. These studies suggest that long-distance seed dispersal mechanisms play a critical role in maintaining genetic links between physically separated populations.

Furthermore, the study acknowledges the limitation of its sampling size, indicating a need for broader sampling to accurately capture the genetic diversity of *C. hainanense*. The importance of extensive sampling in population genetics studies for a more accurate understanding of genetic diversity and structure^26^. Expanding the sampling range could uncover additional layers of genetic variation within the species, providing a more comprehensive picture of its population dynamics.

The potential influence of human activities, such as the movement of seeds or plants, is another aspect that cannot be ignored. Human-mediated gene flow can significantly impact the genetic structure of plant populations^9^. This factor might have contributed to the observed genetic proximity between the Jianfengling and Baishaling populations.

Additionally, small or isolated populations are more susceptible to the effects of genetic drift and founder effects^26^. These phenomena can result in complex genetic patterns, sometimes leading to closely related populations that are geographically distant. To gain a deeper understanding of the genetic structure and evolutionary history of *C. hainanense*, further research is warranted. This research should include an expanded sampling strategy and a focus on understanding the mechanisms of seed dispersal and the historical patterns of gene flow. Such comprehensive studies are crucial for informing effective conservation strategies and ensuring the long-term survival of *C. hainanense*.

### Conservation and management strategies

The findings of this study hold significant implications for the conservation and management of critically endangered *C. hainanense* populations within fragmented habitats in Hainan. Based on the observed patterns of genetic diversity, population structure, and inbreeding, the following conservation and management strategies are recommended:

1. *Habitat protection and restoration* Prioritize the preservation of existing habitats and restore degraded areas to improve habitat quality and connectivity. Enhancing connectivity between fragmented habitats will facilitate gene flow among *C. hainanense* populations, promoting genetic diversity and reducing the risk of inbreeding^32^.
2. *Assisted gene flow and population augmentation* Introduce individuals from genetically diverse populations into small, isolated populations to increase genetic diversity and reduce inbreeding. This approach should be undertaken cautiously, considering potential ecological and genetic risks, such as outbreeding depression and disruption of local adaptations.
3. *In-situ conservation* Efforts to protect the natural habitats of *C. hainanense* should be prioritized. This includes maintaining or establishing protected areas, enforcing regulations to prevent habitat destruction, and promoting sustainable land use practices.
4. *Ex situ conservation* Establish ex situ conservation programs, such as seed banks and living collections in botanical gardens, to preserve the genetic diversity of *C. hainanense*. These efforts can serve as a genetic reservoir for potential reintroduction or population augmentation initiatives in the future.
5. *Monitoring and adaptive management* Implement long-term monitoring programs to track changes in genetic diversity, population structure, and habitat conditions. Utilize the collected data to inform adaptive management strategies, ensuring the conservation efforts remain effective and responsive to emerging threats or changing circumstances.
6. *Community engagement and education* Involve local communities in conservation efforts by raising awareness about the importance of preserving *C. hainanense* and its habitat. Promote sustainable land use practices and develop community-based conservation initiatives to empower local stakeholders in the protection and restoration of the species’ habitat.
7. *Legal protection and policy development* Strengthen the legal protection status of *C. hainanense* and its habitat by incorporating the species into national and regional conservation plans. Develop and enforce policies that minimize habitat destruction, such as regulating land-use change, deforestation, and infrastructure development within the species’ range.
8. *Collaborative research and information sharing* Foster collaboration among researchers, conservation practitioners, and policymakers to facilitate the exchange of knowledge, data, and best practices in the conservation of *C. hainanense*. Encourage interdisciplinary research that integrates genetics, ecology, and social science to develop comprehensive conservation strategies.
9. *Climate change adaptation* Consider the potential impacts of climate change on *C. hainanense* populations and their habitats. Develop proactive conservation measures that enhance the species’ resilience to climate change, such as assisted migration, habitat restoration in areas with suitable future climatic conditions, and incorporation of climate change projections into spatial conservation planning.

By implementing these conservation and management strategies, we can contribute to the preservation and restoration of the critically endangered *C. hainanense* and its fragmented habitats in Hainan, while also informing efforts to protect other endangered plant species facing similar challenges.

The low genetic diversity and weak differentiation among populations underscore the vulnerability of *C. hainanense* to environmental changes and stochastic events. Conservation efforts should focus on protecting existing habitats, promoting gene flow between fragmented populations, and possibly enhancing genetic diversity through conservation strategies like managed breeding or transplantation. Understanding the genetic structure of *C. hainanense* provides a framework for similar conservation approaches in other endangered species within fragmented landscapes.

Wild plants with extremely small populations are usually highly inbred. The characteristics of low transmission diversity and high frequency of genetic drift need to be confirmed. Genetic rescue increases genetic diversity, improves fitness, and enhances adaptation to maintain the species’ long-term survival^33^. Instead of increasing the number of surviving individuals by collecting inbred seeds or asexual cuttings of *C. hainanense*, researchers should focus on designing artificial hybridization strategies to reduce inbred offspring. One of the measures to protect the *C. hainanense* population is to set up multiple protection sites to protect natural populations and their surrounding habitats. The second is to strengthen the gene flow between populations, such as constructing artificial ex-situ protection populations that should be obtained from as many different populations as possible. During ex-situ conservation, the exchange of seeds and seedlings between populations should be increased to create conditions for gene exchange and recombination artificially.

### Limitations and future research directions

Despite the valuable insights generated from this study, certain limitations and future research directions should be acknowledged:

1. *Sample size and representation* The limited number of samples (35) and populations (six distinct cohort groups) included in this study may not entirely capture the full extent of genetic diversity and population structure of *C. hainanense*. Future studies should aim to increase sample sizes and cover a broader range of fragmented habitats to provide a more comprehensive understanding.
2. *Gene flow and landscape connectivity* This study did not investigate the gene flow and landscape connectivity among the fragmented habitats. Understanding how landscape features influence gene flow between populations can provide crucial information for developing conservation corridors and habitat restoration strategies. Future research should incorporate landscape genetic approaches to investigate the impact of habitat fragmentation on gene flow in *C. hainanense* populations.
3. *Functional genetic diversity* The assessment of genetic diversity in this study primarily focused on neutral genetic markers (SNPs). However, functional genetic diversity, which reflects the genetic variation underlying ecologically important traits, is also crucial for species survival and adaptation. Future studies should explore functional genetic diversity by incorporating candidate genes or whole-genome sequencing approaches.
4. *Long-term monitoring and adaptive management* The dynamics of genetic diversity and population structure can change over time due to various factors, such as climate change, anthropogenic disturbances, and random genetic drift. Long-term monitoring of *C. hainanense* populations is essential for detecting changes in genetic diversity and adjusting conservation strategies accordingly.

## Conclusion

This study offers critical insights into the genetic diversity and population structure of *C. hainanense*, a critically endangered species, within fragmented habitats in Hainan Province. Our research significantly contributes to the understanding of how habitat fragmentation affects genetic diversity in plant populations. This understanding is vital for guiding conservation strategies for *C. hainanense* and other endangered plant species in similar fragmented ecosystems. The genetic diversity within *C. hainanense* was found to be low. This low diversity is a concern as it may affect the species’ ability to adapt to environmental changes and could increase the risk of extinction. The heterozygosity of the six examined populations of *C. hainanense* ranged from 10.79 to 14.55%, with an average heterozygosity of 13.15%. These values are indicative of the genetic variation within the species. The relatively narrow range of heterozygosity values across the populations suggests a uniformly low level of genetic diversity across the different habitats. We categorized the six populations of *C. hainanense* into two distinct groups: one consisting of the Diaoluoshan and Baishaling populations, and the other comprising the Wuzhishan, Huishan, Bawangling, and Jianfengling populations. This classification may reflect historical connectivity and genetic flow between these groups. Our findings show that genetic differentiation among *C. hainanense* populations is weak. This could be attributed to historical gene flow between populations or a recent common ancestry.

### Supplementary Information

Supplementary Information.

## Data Availability

The data that support the findings of this study are openly available in the Science Data Bank at https://www.scidb.cn/s/6ZZNn2.
