# Supplementary material for: Assessing genetic diversity in critically endangered Chieniodendron hainanense populations within fragmented habitats in Hainan
Source: Sci Rep. 2024 Mar 24;14:6988. doi: 10.1038/s41598-024-56630-0 (PMC10961303; doi:10.1038/s41598-024-56630-0)
Supplement: Supplementary file 1 — Supplementary Information. [file 41598_2024_56630_MOESM1_ESM.docx]

**Description of the materials used and the GBS dataset**

| Sample | Raw data（bp） | Raw data | Clean data（bp） | Clean data | Effective data (%) | Q20 (%) | Q30 (%) | GC (%) |
| --- | --- | --- | --- | --- | --- | --- | --- | --- |
| BWL_1 | 8889204 | 1.33G | 8790448 | 1.31G | 98.62 | 96.64 | 90.84 | 40.71 |
| BWL_2 | 9549572 | 1.43G | 9468076 | 1.42G | 98.89 | 96.88 | 91.32 | 40.50 |
| BWL_3 | 7803624 | 1.17G | 7720330 | 1.15G | 98.66 | 96.64 | 90.86 | 40.62 |
| BWL_4 | 11810220 | 1.77G | 11691152 | 1.75G | 98.76 | 96.86 | 91.31 | 40.45 |
| BWL_5 | 9345482 | 1.40G | 9257722 | 1.39G | 98.83 | 96.85 | 91.27 | 40.22 |
| BWL_6 | 10039148 | 1.51G | 9929304 | 1.49G | 98.69 | 96.79 | 91.17 | 40.46 |
| BWL_7 | 12080396 | 1.81G | 11953338 | 1.79G | 98.73 | 96.63 | 90.79 | 40.58 |
| BWL_8 | 7625154 | 1.14G | 7549916 | 1.13G | 98.75 | 96.85 | 91.29 | 40.52 |
| JFL_1 | 10316262 | 1.55G | 10212558 | 1.53G | 98.77 | 96.72 | 91.00 | 40.47 |
| JFL_2 | 7804798 | 1.17G | 7718160 | 1.15G | 98.60 | 96.52 | 90.61 | 40.81 |
| JFL_3 | 11219492 | 1.68G | 11103264 | 1.66G | 98.72 | 96.81 | 91.15 | 40.73 |
| JFL_4 | 8774572 | 1.32G | 8672816 | 1.30G | 98.63 | 96.61 | 90.80 | 40.41 |
| JFL_5 | 4550192 | 0.68G | 4506960 | 0.67G | 98.66 | 96.70 | 90.99 | 41.15 |
| DLS_1 | 6437982 | 0.97G | 6370380 | 0.95G | 98.67 | 96.53 | 90.62 | 40.83 |
| DLS_2 | 9621628 | 1.44G | 9534270 | 1.43G | 98.85 | 96.94 | 91.44 | 40.24 |
| DLS_3 | 9154942 | 1.37G | 9071040 | 1.36G | 98.74 | 96.87 | 91.34 | 40.85 |
| DLS_4 | 8780780 | 1.32G | 8704170 | 1.30G | 98.89 | 96.58 | 90.65 | 40.34 |
| DLS_5 | 9331598 | 1.40G | 9241430 | 1.38G | 98.80 | 96.21 | 89.92 | 40.52 |
| DLS_6 | 6398210 | 0.96G | 6355132 | 0.95G | 98.99 | 97.12 | 91.81 | 40.54 |
| DLS_7 | 10561018 | 1.58G | 10452984 | 1.56G | 98.75 | 96.53 | 90.58 | 40.78 |
| DLS_8 | 7790468 | 1.17G | 7711004 | 1.15G | 98.73 | 96.55 | 90.66 | 40.87 |
| DLS_9 | 11100134 | 1.67G | 10983862 | 1.64G | 98.72 | 96.62 | 90.81 | 40.92 |
| DLS_10 | 15223484 | 2.28G | 15095270 | 2.26G | 98.96 | 96.78 | 91.08 | 40.59 |
| HS_1 | 10318662 | 1.55G | 10230010 | 1.53G | 98.91 | 96.67 | 90.85 | 40.52 |
| HS_2 | 9598258 | 1.44G | 9493420 | 1.42G | 98.65 | 96.54 | 90.66 | 41.04 |
| HS_3 | 8071266 | 1.21G | 7978090 | 1.19G | 98.51 | 96.40 | 90.39 | 40.76 |
| HS_4 | 6031464 | 0.90G | 5965206 | 0.89G | 98.50 | 96.67 | 90.94 | 41.34 |
| HS_5 | 11441984 | 1.72G | 11325736 | 1.69G | 98.75 | 96.81 | 91.19 | 40.48 |
| HS_6 | 8636182 | 1.30G | 8551474 | 1.28G | 98.77 | 96.99 | 91.62 | 40.62 |
| WZS_1 | 8937320 | 1.34G | 8841150 | 1.32G | 98.64 | 96.63 | 90.82 | 39.99 |
| WZS_2 | 8653196 | 1.30G | 8572226 | 1.28G | 98.80 | 96.99 | 91.58 | 40.11 |
| WZS_3 | 14758962 | 2.21G | 14618492 | 2.19G | 98.88 | 96.90 | 91.36 | 40.49 |
| BSL_1 | 9710790 | 1.46G | 9610614 | 1.44G | 98.75 | 96.69 | 90.93 | 40.47 |
| BSL_2 | 8528878 | 1.28G | 8442784 | 1.26G | 98.74 | 96.54 | 90.60 | 40.25 |
| BSL_3 | 8876886 | 1.33G | 8780592 | 1.31G | 98.70 | 96.64 | 90.83 | 40.74 |
